# Mesenchymal Stem Cell-Derived Exosomes Ameliorate Delayed Neurocognitive Recovery in Aged Mice by Inhibiting Hippocampus Ferroptosis via Activating SIRT1/Nrf2/HO-1 Signaling Pathway

**DOI:** 10.1155/2022/3593294

**Published:** 2022-09-30

**Authors:** Jie Liu, Jingyao Huang, Zhenjiang Zhang, Rui Zhang, Qijuan Sun, Zhihao Zhang, Yongxin Liu, Baoyu Ma

**Affiliations:** ^1^Shandong Provincial Medicine and Health Key Laboratory of Clinical Anesthesia, School of Anesthesiology, Weifang Medical University, Weifang, China; ^2^Department of Thoracic Surgery, Weifang People's Hospital, Weifang, China

## Abstract

Delayed neurocognitive recovery (dNCR) is a prevalent perioperative neurological complication in older patients and has common characteristics such as acute cognitive dysfunction, impaired memory, and inattention. Mesenchymal stem cell-derived exosomes (MSCs-Exo) are enclosed by a lipid bilayer contain proteins, DNA, miRNA, and other components, which are important mediators of intercellular communication. It has been reported that exosomes could play an important role in the treatment of neurodegenerative diseases, nerve injury, and other neurological diseases. In this study, we examined the effects of MSCs-Exo on dNCR aged mice after exploratory laparotomy and evaluated their potential regulatory mechanisms. We found that MSCs-Exo treatment ameliorated cognitive impairment in dNCR aged mice. MSCs-Exo inhibit hippocampus ferroptosis and increase the expression of silent information regulator 1 (SIRT1), factor nuclear factor-erythroid 2-related factor 2 (Nrf2), and heme oxygenase-1 (HO-1) in dNCR aged mice. Interestingly, the above effects of MSCs-Exo on dNCR aged mice were abolished by SIRT1 selective inhibitor EX-527. In conclusion, these findings indicated that MSCs-Exo can ameliorate cognitive impairment by inhibiting hippocampus ferroptosis in dNCR aged mice via activating SIRT1/Nrf2/HO-1 signaling pathway, providing a potential avenue for the treatment of dNCR.

## 1. Introduction

Delayed neurocognitive recovery (dNCR), which is described by cognitive impairment, is a prevalent perioperative neurological complication in patients within 30 days following surgery, especially in older patients [1]. dNCR is clinically characterized by a sharp decline in cognitive function, including learning, memory, information processing, and attention. And the development of dNCR can increase hospital costs, prolong hospitalization, and increase morbidity and mortality [2], affecting the life quality of patients and aggravating the economic burden of society. Despite significant advances in surgical techniques and anesthesia management, the incidence of dNCR remains at 18-45% [3]. Many studies have attempted to explain the pathogenesis of dNCR from receptor changes, signaling pathways, cytokines, and other aspects, but no ideal therapeutic strategy has been found for the prevention and treatment of dNCR [4]. Therefore, to explore the prevention and treatment of dNCR has become an urgent and important issue in global medical research [5].

Growing evidence demonstrated that the overproduction of reactive oxygen species (ROS), mitochondrial damage, neuroinflammation, and abnormal homocysteine metabolism are crucial for the development of neurological diseases, such as ischemic stroke and cognitive impairment [6–10]. It was reported that ferroptosis could participate in oxidative stress response, mitochondrial dysfunction, neuroinflammation, and abnormal homocysteine metabolism [11–13]. Ferroptosis, a new form of cell death, is characterized by the accumulation of a large amount of iron and lipid peroxidation in cells and mitochondrial shrinkage [14]. It is associated with various diseases, including cancer, acute kidney injury, intracerebral hemorrhage, traumatic brain injury, and various neurological diseases such as Parkinson's disease, Huntington's disease, and Alzheimer's disease [15–18]. Ferroptosis is mediated by glutathione peroxidase 4 (GPX4) [19], which can prevent cell death from oxidative damage [20]. Given the effect of oxidative damage in surgery-induced cognitive impairment [7, 21], we speculate that ferroptosis may participate in dNCR aged mice.

Exosomes are nanosized vesicles (30-150 nm diameter) released by different types of cells [22]. Exosomes contain proteins, DNA, miRNA, lncRNA, and other components, which play a significant role in the interaction between cells and extracellular microenvironment [23]. Numerous studies have found that exosomes could play an important role in the treatment of neurodegenerative diseases, nerve injury, and other neurological diseases [24]. And mesenchymal stem cell-derived exosomes (MSCs-Exo) can alleviate early brain injury and improve cognitive function after subarachnoid hemorrhage [25]. Exosomes have excellent antioxidant properties by stimulating some antioxidant enzymes, such as glutathione peroxidase (GPX) [20]. Moreover, exosomes were reported to inhibit ferroptosis and ameliorate ROS-mediated neuronal cell injury [26]. Hence, MSCs-Exo may inhibit ferroptosis to ameliorate cognitive function in dNCR mice.

Silent information regulator 1 (SIRT1) is a nicotinamide adenosine dinucleotide- (NAD-) dependent class III histone deacetylase [27]. It is widely expressed in various organs, and in the brain, the expression level of SIRT1 is higher in the hippocampus and hypothalamus [28]. SIRT1 in neural system plays an important role in regulating normal brain functions such as plasticity and memory [29]. Recently, SIRT1 was found to play an important role in Alzheimer's disease and Parkinson's disease [30, 31]. SIRT1 is regarded as a protector of the cells against oxidative stress injury and lipid peroxidation by mediating the expression of factor nuclear factor-erythroid 2-related factor 2 (Nrf2) and its downstream target heme oxygenase-1 (HO-1) [32]. Nrf2 can play a key role in neuronal resistance to oxidative stress by mediating HO-1 and alleviating diabetes-associated cognitive impairment [33]. In addition, Song and Long found that Nrf2 and HO-1 could participate in the synthesis of GPX4 [34]. And these two factors could play a critical role in the development of ferroptosis [35, 36]. It is reported that SIRT1 could protect against doxorubicin-induced ferroptosis in cardiomyopathy via the activation of Nrf2 [37]. Furthermore, exosomes can ameliorate ischemic brain injury and exert neuroprotective effects by activating SIRT1 [38].

Based on these promising findings, the present study is aimed at evaluating the effects of MSCs-Exo in model of dNCR and at determining if MSCs-Exo ameliorate cognitive impairment by inhibiting ferroptosis via activating SIRT1/Nrf2/HO-1 pathway, as well as provide potential theoretical and experimental evidence for the treatments of dNCR patients, identifying potential therapeutic targets for clinical treatment.

## 2. Materials and Methods

### 2.1. MSCs-Exo Isolation and Characterization

Mouse bone marrow-derived MSCs were purchased from the Cyagen Biosciences Inc. (Santa Clara, CA, USA) and cultured with a Dulbecco's Modified Eagle's Medium (DMEM) (Solarbio, Beijing, China) containing 10% fetal bovine serum (FBS) (Gibco, Shanghai, China) and 1% penicillin-streptomycin solution (Solarbio, Beijing, China). For MSCs-Exo isolation, when the cells reached 70%-80% confluence, conventional culture medium was replaced by that containing 10% exosome-free FBS (Gibco, Shanghai, China). Following an additional 48 hours of culturing, MSCs-Exo were isolated from cell culture medium by differential centrifugation method using Exo Easy Maxi Kit (QIAGEN, Germantown, MD, USA). After centrifuging, the MSCs-Exo were carefully resuspended in PBS and used immediately or stored at -80° C.

The morphology of MSCs-Exo was observed using transmission electron microscope (TEM) (HT7700-SS, HITACHI, Japan). The characteristic surface marker protein CD63 of MSCs-Exo was analyzed by Western blot. The size distribution of MSCs-Exo was measured by Nanoparticle Tracking Analyzer (Malvern Instruments Ltd., Malvern, UK). The concentration of MSCs-Exo was determined by using bicinchoninic acid assay (BCA) Protein Assay Kit (CWBIO, Beijing, China).

### 2.2. MSCs-Exo Tracing and Immunofluorescence Assay

MSCs-Exo were dyed with PKH26 (Sigma-Aldrich, MO, USA) according to the manufacturer's protocol. In brief, MSCs-Exo were incubated with 4 *μ*l PKH26 diluted in 2 ml Diluent C at room temperature for 15 minutes; then, 2 ml of exosome-free medium was added to stop the labeling reaction. After centrifugation at 100,000 × *g* for 1 hour, the labeled MSCs-Exo were resuspended with PBS before administration.

After the 24-hour MSCs-Exo treatment, the mice were perfused with saline followed by 4% ice-cold paraformaldehyde in PBS under deep anesthesia, and the hemispheres of the brain were dissected and washed with PBS and then fixed at 4°C for 24 hours. The tissues were dehydrated in 30% sucrose solution; when the tissue sank into the bottom, the brains were sliced into 8 *μ*m slices with a cryostat (Leica CM1860 UV, Germany) and placed on adhesive slides and washed with PBS for three times. Finally, the sections were stained with 4,6-diamidino-2-phenylindole (DAPI) at room temperature for 10 minutes and washed with PBS for three times and then captured under a fluorescence microscope (Olympus, Tokyo, Japan).

### 2.3. Animals

Eighteen-month-old specific pathogen-free male C57BL/6 mice (body weight 35–40 g) were purchased from the animal center of Weifang Medical University (Weifang, China). All mice were housed in a room under controlled temperature and humidity conditions, with a 12-hour light/dark cycle, and had free access to food and water. The mice were acclimatized to the laboratory environment for 7 days before the studies. Animal handling and experimental procedures were approved by Committee on the Ethics of Animal Experiments of Weifang Medical University (Weifang, China).

### 2.4. Establishment of Animal Models

To establish the model of dNCR, an exploratory laparotomy was performed using aseptic procedures as previously described [39]. Briefly, the mice were deeply anesthetized with 2.5% isoflurane; their abdomen regions were shaved and sterilized with iodophor and ethanol. The abdomen was exposed by a 1-1.5 cm midline abdomen vertical incision and then softly to explore the viscera, including the liver, kidney, intestines, and musculature by the sterile swab soaked in saline. Next, 6 cm of intestine was exposed outside the abdominal cavity and rubbed for 30 seconds, and the operation lasted 30 minutes. Subsequently, the incision was closed from the peritoneal muscles to the skin using sterile 5-0 sutures and sterilized with iodophor for three times. After the operation, the mice were subcutaneous administered 1 ml saline for supplementation of liquid volume. Finally, they were placed on a heat blanket in a chamber until recovery, and lidocaine hydrochloride gel was applied to the incision to relieve the pain.

### 2.5. Experimental Design

All experimental mice were randomly divided into the following groups (5 mice/group).

Aged mice were used to evaluate the distribution of PKH26-labeled MSCs-Exo administered. Twenty-four hours after MSCs-Exo administration, all mice were decapitated under deep anesthesia and fixed with 4% paraformaldehyde before frozen sectioning.

To explore the effect of surgery on cognitive function in aged mice and whether ferroptosis was present in mice hippocampus, the mice were randomly divided into 3 groups. (1) Sham group: the mice were anesthetized, and their abdomen regions were shaved and sterilized with iodophor and ethanol, but not treated with exploratory laparotomy. And the mice were intraperitoneal injected with corresponding vehicle; (2) dNCR group: the mice were anesthetized and received exploratory laparotomy. And the mice were intraperitoneal injected with corresponding vehicle; (3) dNCR+Fer-1 (ferrostatin-1, a ferroptosis inhibitor) group: the mice were injected with Fer-1 1 hour before surgery through the tail vein (1.5 mg/kg, 1% DMSO diluted in sterile saline, Sigma-Aldrich, MO, USA). The doses of Fer-1 were administered, as described previously [40].

To explore the protective effect of MSCs-Exo in dNCR mice, the mice were randomly divided into 4 groups: (1) Sham group; (2) dNCR group; (3) dNCR+MSCs-Exo (50 *μ*g/mouse) group: the mice were injected with MSCs-Exo 1 hour before surgery through the tail vein; and (4) dNCR+Fer-1 group.

**Figure 1:**
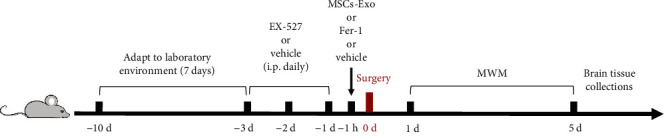
Schematic timeline of the experimental procedure. The mice were acclimatized to the laboratory environment for 7 days before the studies. The EX-527 or corresponding vehicle was injected daily for 3 days before surgery; the MSCs-Exo, or Fer-1, or corresponding vehicle was administered 1 hour before surgery. Next, the behavioral tests were performed began at the 24 hours after the surgery to verify the success of model and the influence of MSCs-Exo on cognitive function in dNCR aged mice. In the end, the mice were decapitated after the behavioral tests, and the brain tissues were collected for subsequent experiments.

To explore the role of SIRT1 played on the protective effect of MSCs-Exo in dNCR mice, the mice were randomly divided into 5 groups: (1) Sham group; (2) dNCR group; (3) dNCR+MSCs-Exo group; (4) dNCR+MSCs-Exo + EX-527 (a selective SIRT1 inhibitor [41, 42]) group: the mice were intraperitoneal injected with EX-527 daily for 3 days before surgery (5 mg/kg, 1% DMSO diluted in sterile saline, Selleck, Houston, TX, USA) and injected with MSCs-Exo 1 hour before surgery through the tail vein; and (5) dNCR+EX-527 group: the mice were intraperitoneal injected with EX-527 daily for 3 days before surgery. The doses of EX-527 were chosen based on earlier reports that EX-527 significantly inhibited the expression of SIRT1 [43]. The experimental timeline was shown in Figure 1; the EX-527 or corresponding vehicle was injected daily for 3 days before surgery; the MSCs-Exo, or Fer-1, or corresponding vehicle was administered 1 hour before surgery. Next, the behavioral tests were performed began at 24 hours after the surgery to verify the success of model and the influence of MSCs-Exo on cognitive function in dNCR aged mice. Eventually, after drug treatment and behavioral tests, the mice were killed for subsequent experiments.

### 2.6. Tail Vein Injection

One hour before surgery, the mouse was taken from the housing cage and introduced onto the tail vein injection device. The injection site of the tail was wiped with a 75% alcohol cotton ball. The needle of a 1 ml syringe was inserted into the skin in a 10-15° angle about 2-4 mm to penetrate the tail vein, and 50 *μ*g MSCs-Exo solution was slowly injected. Then, the needle was removed quickly, and the injection site was pressed firmly to prevent the backflow of the injected drugs and/or blood. Finally, the mouse was returned to its cage and monitored for at least 5 minutes to ensure the mouse has no further bleeding.

### 2.7. Morris Water Maze Test

Morris water maze (MWM), which is a hippocampus-dependent spatial learning and memory test for rodents, was performed as described previously with minor modifications [44]. The MWM consisted of a round steel pool that was 125 cm in diameter, 60 cm in height, and filled with white water maintained at 21 ± 1°C. The pool was surrounded by a gray curtain and placed in a quiet room. The maze was divided into four quadrants with an escape platform (10 cm in diameter) immersed 1 cm below the water surface in one target quadrant, and marked objects are suspended from fixed positions in each quadrant. MWM testing began at the 24 hours after surgery and lasted for five days, with the first 4 days being a training period. The mice were released facing the pool rim from different quadrants and given 90 seconds to find the platform, and they were artificially guided to the platform and stayed there for 15 seconds if they failed to find the platform within 90 seconds. A video surveillance system was used to record and track the escape latency of mice (i.e., the time taken from being released in the water to finding the platform). On the 5th day, the mice were subjected to the space exploration experiment. The platform was removed, and the mice were released from the opposite quadrant of the platform and swam freely for 120 seconds and then recorded the number of crossings over the previously hidden platform and the time spent in the specific target quadrant.

### 2.8. Reactive Oxygen Species (ROS) Assays

The levels of ROS from hippocampus tissues were assessed using the dihydroethidium (DHE) fluorescent probe (Sigma-Aldrich, MO, USA), following the previously described protocols [45]. Briefly, frozen brain sections were incubated with 50 *μ*M DHE at room temperature for 1 hour in the dark; the sections were incubated for 10 minutes with DAPI. Then, the images were captured under a fluorescence microscope (Olympus, Tokyo, Japan).

### 2.9. Glutathione (GSH), Malondialdehyde (MDA), and Ferrous Ion (Fe^2+^) Assays

Aged mice were decapitated under deep anesthesia, and the hippocampus was immediately collected. The fresh tissues of hippocampus were perfused with PBS containing heparin to remove blood and clots. After weighing the tissue, it was homogenized in slurry medium. The relative concentration of GSH was detected using GSH Colorimetric Assay Kit (Elabscience, Wuhan, China). The MDA content was detected using an MDA Colorimetric Assay Kit (TBA method) (Elabscience, Wuhan, China) and the determination of Fe^2+^ level using Ferrous Iron Colorimetric Assay Kit (Elabscience, Wuhan, China). All kits were used according to the manufacturer's instructions.

### 2.10. Western Blot Analysis

The hippocampus tissue was quickly collected under deep anesthesia and quickly bathed in a radioimmunoprecipitation assay (RIPA) lysis buffer combined with proteinase inhibitors. Subsequently, the tissue was ground into a suspension and lysed on ice for 30 minutes and then centrifuged at 12,000 rpm in a cold centrifuge at 4°C for 15 minutes. Afterward, the supernatant was carefully transferred into EP tube. Protein concentration was determined by BCA Protein Assay Kit (CWBIO, Beijing, China). The protein lysate was diluted to the same concentration with 5x loading buffer and denatured by boiling. Then, the proteins in each sample were loaded onto 10% sodium dodecyl sulfate-polyacrylamide gel electrophoresis (SDS-PAGE) gels. Electrophoresis was conducted at 80 V for 30 minutes first and, then, at 120 V for 60 minutes and subsequently transferred onto a 0.45 *μ*m polyvinylidene fluoride (PVDF) membrane. The membranes were blocked with 5% skim milk at room temperature for 3 hours and incubated overnight at 4°C with the following primary antibodies: mouse anti-P53 (1 : 1,000 dilution, 60283-2-Ig; Proteintech), rabbit anti-solute carrier family 7 membrane 11 (SLC7A11) (1 : 1,0000 dilution, ab175186; Abcam), rabbit anti-GPX4 (1 : 1,000 dilution, A11243; ABclonal), mouse anti-SIRT1 (1 : 1,000 dilution, 60303-1-Ig; Proteintech), rabbit anti-Nrf2 (1 : 1,000 dilution, ab92946; Abcam), rabbit anti-HO-1 (1 : 1,000 dilution, A19062; ABclonal), and *β*-actin (1 : 1,0000 dilution, AC026; ABclonal). After that, the membranes were washed five times with 0.1 M TBST for 3 minutes each time and incubated with goat anti-rabbit immunoglobulin G (IgG) (H + L) horseradish peroxidase (HRP) (1 : 1,000 dilution, AS014; ABclonal) or goat anti-mouse IgG (H + L) HRP (1 : 5,000 dilution, AS003; ABclonal) at room temperature for 2 hours. The protein bands were visualized with enhanced chemiluminescence (ECL) detection reagents (CWBIO, Beijing, China) and a computerized image analysis system (Tanon 4600, Shanghai, China). The ImageJ software was used to quantify protein blot intensity.

### 2.11. Transmission Electron Microscope (TEM)

In order to study the ultrastructure, the mice were decapitated while under anesthetized; the hippocampus were quickly separated and cut into 1 mm^3^ pieces with a sharp scalpel and soaked immediately in 2.5% cold glutaraldehyde. Next, these tissues were fixed, dehydrated, embedded, solidified, sectioned, and stained. Finally, the ultrastructural characteristics of hippocampus mitochondria were observed under the TEM (HT7700-SS, HITACHI, Japan).

### 2.12. Statistical Analysis

Data were analyzed using the GraphPad Prism software. All data were presented as mean ± SEM. Group differences in the escape latency during the MWM test were analyzed using the two-way analysis of variance (ANOVA). Statistical significance between two groups was analyzed using a two-tailed Student's *t*-test, and statistical significance between multiple groups was analyzed using one-way ANOVA followed by Bonferroni post hoc test. *P* < 0.05 was considered to be statistically significant.

## 3. Results

### 3.1. Surgery Induced Cognitive Impairment and Hippocampus Ferroptosis in Aged Mice

**Figure 2:**
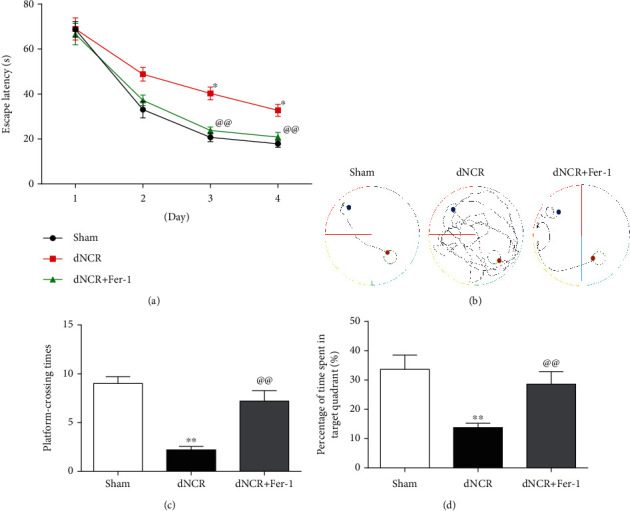
Surgery induced cognitive impairment in aged mice. The mice were randomly divided into 3 groups: Sham group, dNCR group, and dNCR+Fer-1 group. (a) Escape latency during the MWM navigation test. (b) The representative trajectory diagrams of the swimming distance to find the platform (blue dots: starting position; yellow dots: ending position). (c) The platform-crossing times in the MWM test. (d) The time spent in the target quadrant during the MWM test. Data are expressed as mean ± SEM (*n* = 5/group). ^∗^*P* < 0.05 and ^∗∗^*P* < 0.01, Sham vs. dNCR; ^@@^*P* < 0.01, dNCR vs. dNCR+Fer-1. Fer-1: ferrostatin-1, a ferroptosis inhibitor.

Firstly, to verify the reliability of the model, we evaluated the influence of exploratory laparotomy on cognitive function in aged mice using the MWM. When mice were trained for MWM, we found that the mice subjected to surgery showed longer escape latencies compared with the Sham group mice on days 3 and 4 of training (Figure 2(a)). Moreover, the mice in the dNCR group took longer swimming distance of finding the platform than those in the Sham group on days 4 of training (Figure 2(b)). Then, the space exploration experiment was performed on day 5 by removing the platform, releasing the mice from the opposite quadrant of the platform, and recording the number of crossings over the previously hidden platform and the time spent in the specific target quadrant within 120 seconds. We found that the platform-crossing times and the time spent in the target quadrant were significantly decreased in the dNCR group compared with the Sham group (Figures 2(c) and 2(d)). Hence, our results suggested that surgery induced cognitive impairment.

**Figure 3:**
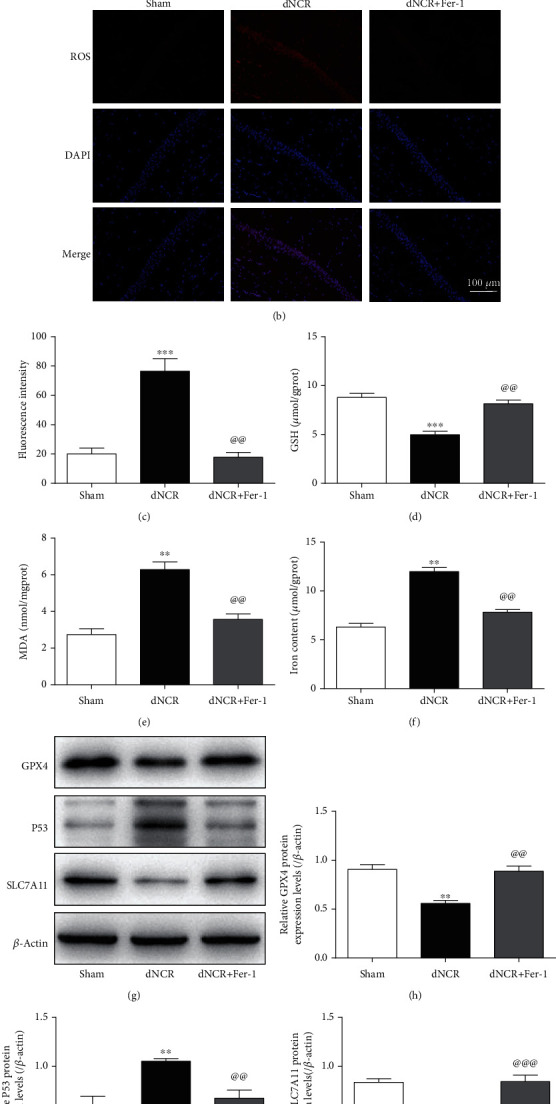
Surgery induced hippocampus ferroptosis in aged mice. The mice were randomly divided into 3 groups: Sham group, dNCR group, and dNCR+Fer-1 group. (a) TEM was employed to detect the ultrastructure of hippocampus in aged mice (bar = 1.0 *μ*m). (b, c) ROS (red fluorescent signal) were detected using DHE staining (bar = 100 *μ*m). (d) The GSH level by GSH Colorimetric Assay Kit. (e) The MDA level by MDA Colorimetric Assay Kit. (f) The Fe^2+^ level by Ferrous Iron Colorimetric Assay Kit. (g–j) GPX4, P53, and SLC7A11 expressions in each group were determined by Western blot. gprot: gram protein; mgprot: milligram protein. Data are expressed as mean ± SEM (*n* = 5/group). ^∗∗^*P* < 0.01 and ^∗∗∗^*P* < 0.001, Sham vs. dNCR; ^@@^*P* < 0.01 and ^@@@^*P* < 0.001, dNCR vs. dNCR+Fer-1. Fer-1: ferrostatin-1, a ferroptosis inhibitor.

Next, the model of dNCR in aged mice was established. Fer-1 was used to determine whether ferroptosis was present in dNCR aged mice hippocampus. The hippocampus tissues of mice were collected to observe the morphology of mitochondria in hippocampus and explore the levels of ROS, GSH, MDA, Fe^2+^, key ferroptosis-related protein GPX4 [46], P53 [47], and SLC7A11 [48]. As shown in Figure 3(a), we observed that compared with mitochondria in the Sham group, the mitochondria morphology in the hippocampus in the dNCR group showed the significant characteristic changes of ferroptosis, including the size of mitochondrial became smaller, the density of double-layer membrane was increased, and the mitochondrial crest was decreased. In addition, compared with the Sham group, GSH level was lower, while ROS, MDA, and Fe^2+^ levels were higher in the dNCR group, and there was no significant difference in GSH, ROS, MDA, and Fe^2+^ levels between the Sham group and Sham+Fer-1 group (Figures 3(b)–3(f)). Meanwhile, the protein expression levels of GPX4 and SLC7A11 were decreased while the protein expression level of P53 was increased in the dNCR group, and there was no significant difference between the Sham group and Sham+Fer-1 group (Figures 3(g)–3(j)). These data suggested that ferroptosis may occur in dNCR aged mice hippocampus.

### 3.2. Phenotypic Identification of Collected MSCs-Exo

**Figure 4:**
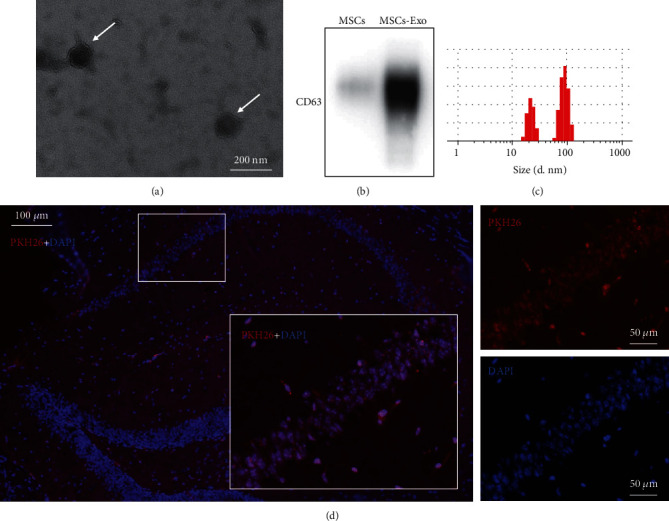
Phenotypic identification of collected MSCs-Exo. (a) Representative electron microscopy image of MSCs-Exo (bar = 200 nm). (b) Representative marker of isolated exosomes detected by Western blot. (c) Size distribution of exosomes determined by Nanoparticle Tracking Analyzer. (d) Representative fluorescence images of brain sections stained; PKH26-dyed exosomes were administered through tail vein into mice.

To investigate the role of MSCs-Exo in dNCR mice, the MSCs-Exo was isolated from culturing medium of MSCs. TEM analysis demonstrated that the particles obtained were round-shaped vesicles with a bilayer membrane structure and with a diameter of approximately 50-100 nm (Figure 4(a)). Western blot showed that the protein marker CD63 was remarkably higher in MSCs-Exo compared with MSC cytoplasm (Figure 4(b)). Nanoparticle tracking analysis demonstrated that the diameters of the most particles were within the range of 60-200 nm, and the peak diameter is 135.7 nm (Figure 4(c)). According to the quantification, the protein concentration of the MSCs-Exo was 0.7570 mg/ml. The above results indicated that the MSCs-Exo have been successfully isolated from MSCs.

To prove that the MSCs-Exo can change gene expression of the hippocampus, firstly, we have demonstrated that the MSCs-Exo could pass through the blood-brain barrier and was absorbed into neurons. We performed MSCs-Exo tracing assays using PKH26-dyed MSC-Exo. After the 24-hour MSCs-Exo treatment, brain sections were obtained. As shown in Figure 4(d), most neurons obtained the PKH26-labeled MSC-Exo.

### 3.3. MSCs-Exo Treatment Ameliorates Cognitive Impairment in dNCR Aged Mice

**Figure 5:**
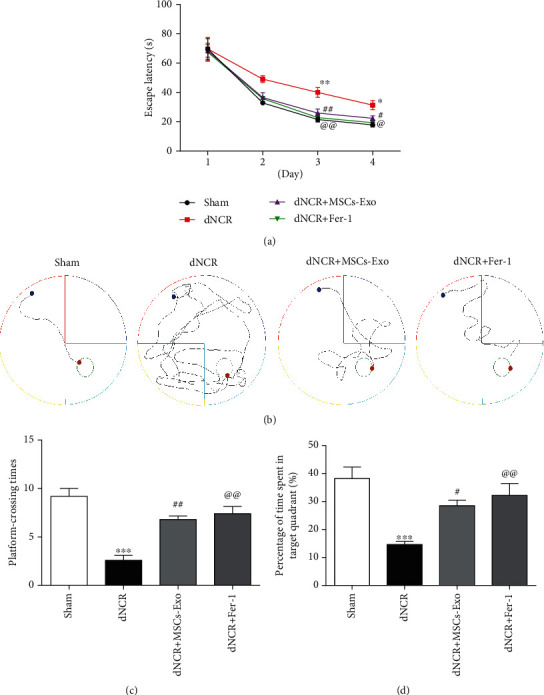
MSCs-Exo treatment ameliorates cognitive impairment in dNCR mice. The mice were randomly divided into 4 groups: Sham group, dNCR group, dNCR+MSCs-Exo group, and dNCR+Fer-1 group. (a) Escape latency during the MWM navigation test. (b) The representative trajectory diagrams of the swimming distance to find the platform (blue dots: starting position; yellow dots: ending position). (c) The platform-crossing times in the MWM test. (d) The time spent in the target quadrant during the MWM test. Data are expressed as mean ± SEM (*n* = 5/group). ^∗^*P* < 0.05, ^∗∗^*P* < 0.01, and ^∗∗∗^*P* < 0.001, Sham vs. dNCR; ^#^*P* < 0.05 and ^##^*P* < 0.01, dNCR vs. dNCR+MSCs-Exo; ^@^*P* < 0.05 and ^@@^*P* < 0.01, dNCR vs. dNCR+Fer-1. Fer-1: ferrostatin-1, a ferroptosis inhibitor.

The hidden platform training and space exploration experiments were performed to test the cognitive function of mice. In Figures 5(a) and 5(b), compared with the dNCR group, the escape latencies on days 3 and 4 of training and the swimming distance of finding the platform on day 4 of training in the dNCR+MSCs-Exo group were significantly reduced. In the space exploration experiment (Figures 5(c) and 5(d)), the platform-crossing times and the time spent in the target quadrant were significantly longer in the dNCR+MSCs-Exo group compared with the dNCR group. Collectively, these findings indicated that MSCs-Exo treatment prevented spatial learning and memory impairments in dNCR aged mice.

### 3.4. MSCs-Exo Inhibit Hippocampus Ferroptosis in dNCR Aged Mice

**Figure 6:**
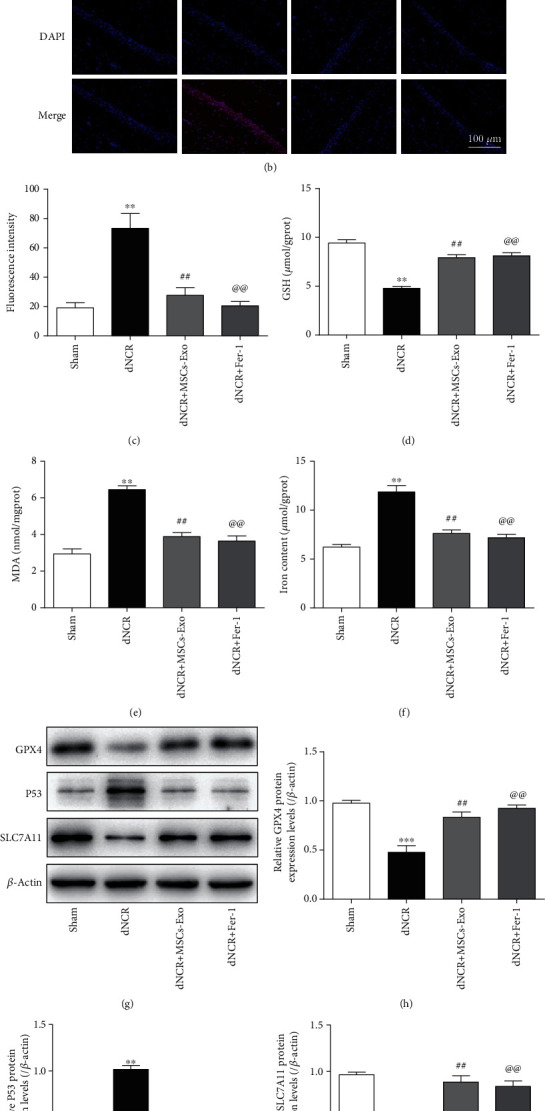
MSCs-Exo inhibit hippocampus ferroptosis in dNCR mice. The mice were randomly divided into 4 groups: Sham group, dNCR group, dNCR+MSCs-Exo group, and dNCR+Fer-1 group. (a) TEM was employed to detect the ultrastructure of hippocampus in aged mice (bar = 1.0 *μ*m). (b, c) ROS (red fluorescent signal) were detected using DHE staining (bar = 100 *μ*m). (d) The GSH level by GSH Colorimetric Assay Kit. (e) The MDA level by MDA Colorimetric Assay Kit. (f) The Fe^2+^ level by Ferrous Iron Colorimetric Assay Kit. (g–j) GPX4, P53, and SLC7A11 expressions in each group were determined by Western blot. gprot: gram protein; mgprot: milligram protein. Data are expressed as mean ± SEM (*n* = 5/group). ^∗∗^*P* < 0.01 and ^∗∗∗^*P* < 0.001, Sham vs. dNCR; ^##^*P* < 0.01, dNCR vs. dNCR+ MSCs-Exo; ^@@^*P* < 0.01, dNCR vs. dNCR+Fer-1. Fer-1: ferrostatin-1, a ferroptosis inhibitor.

We use Fer-1 to further determine whether the protective effect of MSCs-Exo on cognitive impairment is involved in inhibiting ferroptosis. As shown in Figure 6(a), compared with the dNCR group, the size of mitochondria was increased, the density of double-layer membrane was reduced, and the mitochondrial crest was increased in the dNCR+MSCs-Exo group and dNCR+Fer-1 group. Additionally, compared with increased ferroptosis in dNCR group, significantly reduced ROS, MDA, and Fe^2+^ levels combined with elevated GSH level was found in the dNCR+MSCs-Exo group and dNCR+Fer-1 group (Figures 6(b)–6(f)). Meanwhile, the results of Western blot showed that the protein expression levels of GPX4 and SLC7A11 were increased, while the protein expression level of P53 was reduced in the dNCR+MSCs-Exo group and dNCR+Fer-1 group compared with the dNCR group (Figures 6(g)–6(j)). These data suggested that MSCs-Exo inhibited hippocampus ferroptosis in dNCR aged mice.

### 3.5. The Effect of MSCs-Exo on Hippocampus Ferroptosis in dNCR Mice Is Mediated by the Nrf2/HO-1 Signaling Pathway

**Figure 7:**
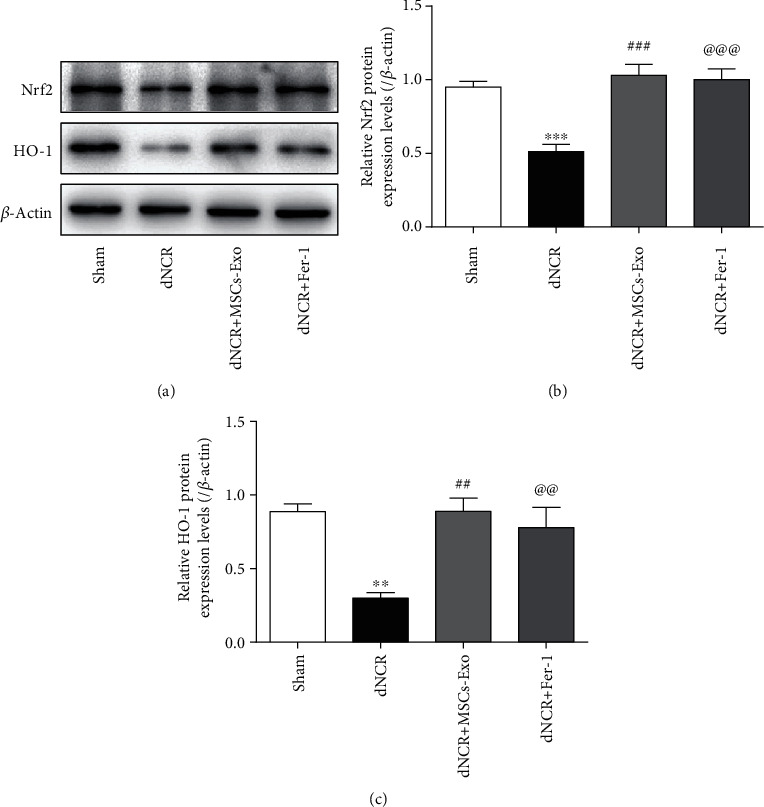
The effect of MSCs-Exo on hippocampus ferroptosis in dNCR mice is mediated by the Nrf2/HO-1 signaling pathway. The mice were randomly divided into 4 groups: Sham group, dNCR group, dNCR+MSCs-Exo group, and dNCR+Fer-1 group. (a–c) Nrf2 and HO-1 expressions in each group were determined by Western blot. Data are expressed as mean ± SEM (*n* = 5/group). ^∗∗^*P* < 0.01 and ^∗∗∗^*P* < 0.001, Sham vs. dNCR; ^##^*P* < 0.01 and ^###^*P* < 0.001, dNCR vs. dNCR+ MSCs-Exo; ^@@^*P* < 0.01 and ^@@@^*P* < 0.001, dNCR vs. dNCR+Fer-1. Fer-1: ferrostatin-1, a ferroptosis inhibitor.

Ferroptosis is characterized by the massive accumulation of fatal intracellular lipid peroxide when the antioxidant capacity of cells decreases [34]. The transcription factor Nrf2 is a well-known transcription factor that plays a key role in against oxidative stress. A previous study demonstrated that Nrf2-regulated signaling pathway diminished anesthesia-induced memory impairment by inhibiting oxidative stress [49]. Thus, we determined whether the effect of MSCs-Exo on ferroptosis in dNCR aged mice is mediated by the Nrf2 and HO-1 pathways. According to Western blot analysis (Figures 7(a)–7(c)), the results suggested that MSCs-Exo and Fer-1 significantly increased the expression of Nrf2 compared with that in the dNCR group. Additionally, HO-1, the downstream component of the Nrf2 pathway, was upregulated by treatment with MSCs-Exo and Fer-1. Thus, we suggested that MSCs-Exo mitigate the ferroptosis in dNCR mice via activating Nrf2/HO-1 pathway.

### 3.6. MSCs-Exo Alleviated Cognitive Impairment and Hippocampus Ferroptosis of dNCR Aged Mice in a SIRT1-Dependent Manner

**Figure 8:**
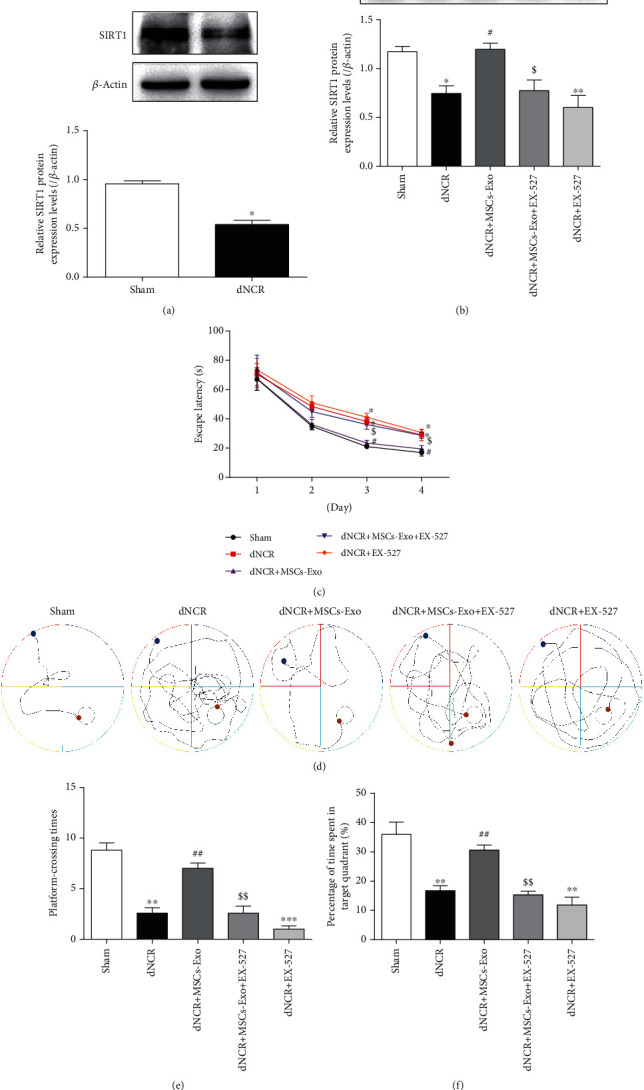
MSCs-Exo alleviated cognitive impairment of dNCR mice in a SIRT1-dependent manner. The mice were randomly divided into 5 groups: Sham group, dNCR group, dNCR+MSCs-Exo group, dNCR+MSCs-Exo + EX-527 group, and dNCR+EX-527 group. (a, b) SIRT1 expression in each group was determined by Western blot. (c) Escape latency during the MWM navigation test. (d) The representative trajectory diagrams of the swimming distance to find the platform (blue dots: starting position; yellow dots: ending position). (e) The platform-crossing times in the MWM test. (f) The time spent in the target quadrant during the MWM test. Data are expressed as mean ± SEM (*n* = 5/group). ^∗^*P* < 0.05, ^∗∗^*P* < 0.01, and ^∗∗∗^*P* < 0.001, Sham vs. dNCR or Sham vs. dNCR+EX-527; ^#^*P* < 0.05 and ^##^*P* < 0.01, dNCR vs. dNCR+MSCs-Exo; ^$^*P* < 0.05 and ^$$^*P* < 0.01, dNCR+MSCs-Exo vs. dNCR+MSCs-Exo + EX-527. EX-527: a specific SIRT1 inhibitor.

SIRT1 is considered an effective protector of the cells against oxidative insult acting by targeting Nrf2 to regulate the expression of HO-1 [19]. The data of Figure 8(a) showed that surgery induced a decrease in the protein expression of SIRT1 compared with that in the Sham group. To explore whether the effect of MSCs-Exo is SIRT1 dependent, we pretreated mice with EX-527, a specific SIRT1 inhibitor. The data of Figure 8(b) showed that the protein expression level of SIRT1 was downregulated by surgery or EX-527 and restored by MSCs-Exo, which indicated that the effect of MSCs-Exo was mediated by regulation of SIRT1.

Additionally, the dNCR group and dNCR+EX-527 group exhibited longer escape latencies, longer swimming distance of finding the platform, fewer platform-crossing times, and shorter time spent in the target quadrant than the Sham group mice, and there was no difference between the dNCR group and the dNCR+EX-527 group (Figures 8(c)–8(f)). In contrast, in the dNCR+MSCs-Exo group, the escape latencies on days 3 and 4 of training were shorter, the swimming distance of finding the platform on day 4 of training was longer, the platform-crossing times and the time spent in the target quadrant were significantly increased (Figures 8(c)–8(f)). Interestingly, dNCR+MSCs-Exo + EX-527 treatment significantly affected the MSCs-Exo inhibitory effect on cognition impairment. Administration of EX-527 prior to dNCR+MSCs-Exo treatment abolished the protective effects of MSCs-Exo (Figures 8(c)–8(f)). Pretreated dNCR+MSCs-Exo mice with EX-527 extended their escape latencies and the swimming distance of finding the platform (Figures 8(c) and 8(d)). In the space exploration experiment, the platform-crossing times and the time spent in the target quadrant were significantly shorter in the dNCR+MSCs-Exo + EX-527 group than in the dNCR+MSCs-Exo group (Figures 8(e) and 8(f)). These results indicated that MSCs-Exo treatment was effective in improving spatial learning and memory of dNCR aged mice in a SIRT1-dependent manner.

**Figure 9:**
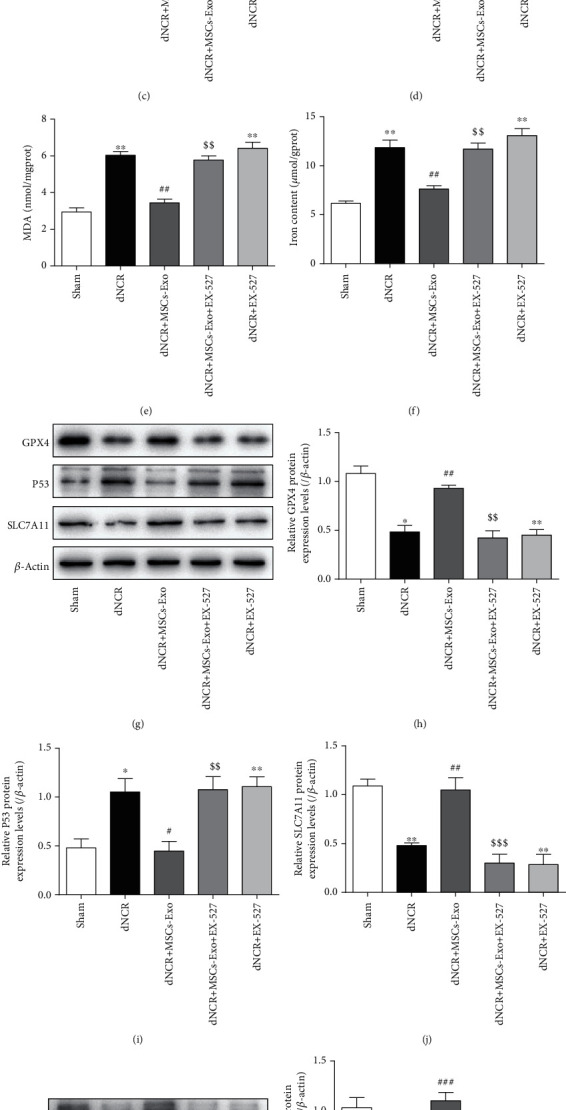
MSCs-Exo alleviated hippocampus ferroptosis of dNCR mice in a SIRT1-dependent manner. The mice were randomly divided into 5 groups: Sham group, dNCR group, dNCR+MSCs-Exo group, dNCR+MSCs-Exo + EX-527 group, and dNCR+EX-527 group. (a) TEM was employed to detect the ultrastructure of hippocampus in aged mice (bar = 1.0 *μ*m). (b, c) ROS (red fluorescent signal) were detected using DHE staining (bar = 100 *μ*m). (d) The GSH level by GSH Colorimetric Assay Kit. (e) The MDA level by MDA Colorimetric Assay Kit. (f) The Fe^2+^ level by Ferrous Iron Colorimetric Assay Kit. (g–j) GPX4, P53, and SLC7A11 expressions in each group were determined by Western blot. (k–m) Nrf2 and HO-1 expressions in each group were determined by Western blot. gprot: gram protein; mgprot: milligram protein. Data are expressed as mean ± SEM (*n* = 5/group). ^∗^*P* < 0.05, ^∗∗^*P* < 0.01, and ^∗∗∗^*P* < 0.001, Sham vs. dNCR or Sham vs. dNCR+EX-527; ^#^*P* < 0.05, ^##^*P* < 0.01, ^###^*P* < 0.001, dNCR vs. dNCR+MSCs-Exo; ^$^*P* < 0.05, ^$$^*P* < 0.01, and ^$$$^*P* < 0.001, dNCR+MSCs-Exo vs. dNCR+MSCs-Exo + EX-527. EX-527: a specific SIRT1 inhibitor.

We then examined the effect of MSCs-Exo-mediated SIRT1 activation on hippocampus ferroptosis in dNCR mice. The results showed that the MSCs-Exo alleviated hippocampus ferroptosis of dNCR aged mice was abolished by EX-527 treatment. Compared with the Sham group, the dNCR group and dNCR+EX-527 group mitochondria observed via TEM had shrunk in size, the double-layer membrane density had reduced, and the mitochondrial crest had decreased or disappeared. In contrast, MSCs-Exo ameliorated ferroptosis-induced mitochondrial morphologic changes, and pretreating dNCR+MSCs-Exo mice with EX-527 the mitochondria morphology in hippocampus showed the characteristic changes of ferroptosis (Figure 9(a)). In addition, compared with the Sham group, the GSH level was lower, while ROS, MDA, and Fe^2+^ levels were higher in the dNCR group and dNCR+EX-527 group (Figures 9(b)–9(f)). Meanwhile, the protein expression levels of GPX4 and SLC7A11 were decreased while the protein expression level of P53 was increased (Figures 9(g)–9(j)). There was no difference between the dNCR group and the dNCR+EX-527 group (Figures 9(b)–9(j)). In contrast, MSCs-Exo treatment increased the levels of GSH, GPX4, and SLC7A11 and decreased the levels of ROS, MDA, Fe^2+^, and P53 which were reversed by EX-527 treatment (Figures 9(b)–9(j)). Furthermore, compared with the dNCR group, we observed that MSCs-Exo administration increased the expression of Nrf2 and HO-1 in the hippocampus, whereas pretreated mice with EX-527 decreased their expression (Figures 9(k)–9(m)). These data suggested that the Nrf2/HO-1 pathway was regulated by SIRT1 to inhibit hippocampus ferroptosis.

Taken together, our results suggested that MSCs-Exo effectively ameliorated the cognitive impairment and inhibited hippocampus ferroptosis in dNCR aged mice through a SIRT1-dependent mechanism.

## 4. Discussion

**Figure 10:**
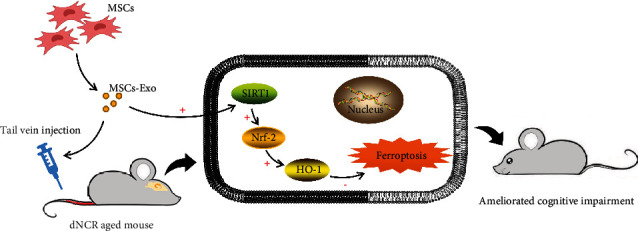
Diagrammatic presentation of the findings from this study. MSCs-Exo upregulates the expression level of SIRT1. SIRT1 restoration leads to an increase in Nrf2 and HO-1. Collectively, this mechanism inhibits ferroptosis induced by surgery so as to alleviate cognitive impairment. MSCs: mesenchymal stem cells; MSCs-Exo: mesenchymal stem cell-derived exosomes; dNCR: delayed neurocognitive recovery; SIRT1: silent information regulator 1; Nrf2: factor nuclear factor-erythroid 2-related factor 2; HO-1: heme oxygenase-1.

The current study was conducted to examine the effects and mechanisms of MSCs-Exo on cognitive dysfunction induced by surgery in aged mice. Here, we found that laparotomy surgery induced cognitive impairment and hippocampus ferroptosis. Exogenous MSCs-Exo could ameliorate cognitive dysfunction by inhibiting ferroptosis. In addition, MSCs-Exo could reverse the expression level of SIRT1 reduced by surgery. And pretreatment specific SIRT1 inhibitor EX-527 abolished improving effects of MSCs-Exo in cognitive impairment and ferroptosis of aged mice after surgery. Moreover, using EX-527 could also block the Nrf2/HO-1 signaling pathway in MSCs-Exo-treated aged mice. The protective effects of MSCs-Exo on cognitive impairment in aged mice were related to its inhibited hippocampus ferroptosis by modulating the activity of SIRT1/Nrf2/HO-1 signaling pathway (Figure 10).

dNCR is a perioperative neurological complication, representing major health concerns for older surgical patients [50], especially experiencing hip-fracture repair and cardiac surgery [51]. dNCR is a main cause of functional impairment and increased morbidity and mortality [1]. Despite increasing investigation of dNCR mechanisms and therapeutic strategies, the current therapies for the management of dNCR are still insufficient. Exosomes are rich in bioactive molecules, including DNA, proteins, mRNAs, and miRNAs, which make exosomes essential for intercellular communication [52]. MSCs-Exo plays an important role in oxidative stress response, neurodegenerative disease, and neurological diseases [53–55]. In our study, the dNCR model of aged mice was established to investigate the protective effect of MSC-Exo. Firstly, we found that an exploratory laparotomy was performed using aseptic procedures caused cognitive dysfunction in aged mice, which is in consistent with previous studies [39]. And MWM tests showed that the mice in the dNCR+MSCs-Exo group spent less time to find the platform and more time in the target quadrant than that of the dNCR group. These results indicated that MSCs-Exo pretreatment could alleviate the cognitive impairment caused by surgery.

Iron is one of the most important minerals, which plays an indispensable role in many physiological and pathological processes of the body [34, 56, 57]. In neuronal system, iron homeostasis is important for brain function including enzyme catalysis, mitochondrial function, myelination, and synaptic plasticity. Dysregulation of iron homeostasis can cause oxidative stress and inflammation, leading to cell damage and ultimately neurological disease [58–60]. Ferroptosis is a programmed cell death process associated with dysregulation of iron homeostasis, which is characterized by iron-dependent lipid peroxidation [61]. Previous studies suggested that ferroptosis was present in intestinal I/R injury and lung I/R injury in mice [62, 63]. And ferroptosis was found an important role in cognitive deficits of neurodegenerative diseases [34, 64]. In our study, we found the increased Fe^2+^ level in hippocampus of dNCR mice. Meanwhile, the levels of ROS, MDA, and P53 were increased, while the levels of GSH, GPX4, and SLC7A11 were decreased in dNCR mice. In addition, we also found that the increased levels of ROS, MDA, Fe^2+^, and P53 and decreased levels of GSH, GPX4, and SLC7A11 in dNCR mice were reversed by Fer-1, suggesting that ferroptosis can participate in the development of dNCR. Song et al. found that MSCs-Exo could attenuate myocardial injury by inhibiting ferroptosis [65]. In recent studies, exosomes could inhibit ferroptosis in neural system and play a protective role in intracerebral hemorrhage [66]. In this study, we found that MSCs-Exo treatment could downregulate the levels of ROS, MDA, Fe^2+^, and P53, while increase the levels of GSH, GPX4, and SLC7A11 in dNCR mice. These results indicated that MSCs-Exo could alleviate the cognitive impairment by mediating hippocampus ferroptosis in dNCR aged mice.

SIRT1, a NAD-dependent deacetylase, plays a positive role in stress responses, cellular metabolism, and aging [67]. SIRT1 in the nervous system can participate in neuroprotection, oxidative stress, inflammatory response, autophagy, and other biological processes [68]. In our study, we found that SIRT1 expression was downregulated in the dNCR mice. Previous studies found that extracellular vesicles, which contain exosomes, could enhance SIRT1 activation, synaptic activity, and rescue cognitive deficits in AD model [69]. And another study suggested that SIRT1 was activated by exosomes to exert protective effect against radiation-induced brain injury [70]. Our results found that exogenous MSCs-Exo could restore the downregulation of SIRT1, suggesting the regulatory role of exosomes in SIRT1 function in dNCR. Furthermore, studies have shown that the bioactive molecules miRNA in exosomes can regulate SIRT1 [71, 72]. However, what component in MSCs-Exo regulate SIRT1 to ameliorate dNCR in aged mice needs to be further explored.

In addition, our results also found that inhibiting SIRT1 activation could result in ferroptosis in dNCR mice. SIRT1 plays an important role in neurodegenerative diseases and cognitive deficits via regulating the levels of Nrf2 and HO-1 [1, 73–76]. The activation of SIRT1 targeting Nrf2/HO-1 pathway activation can alleviate central nervous system inflammation-induced cognitive deficits [73]. Nrf2/HO-1 pathway can play a key role in suppressing ferroptosis in nervous system [34, 77]. Dang et al. revealed that edaravone abolished chronic social defeat stress-induced ferroptosis and ameliorated depressive and anxiety-like behaviors by regulating SIRT1/Nrf2/HO-1/GPX4 pathway [20]. Our study found that the expressions of Nrf2 and HO-1 were increased accompanied by ameliorated dNCR after MSCs-Exo treatment, and SIRT1 inhibitor could inhibit this Nrf2/HO-1 pathway activation, suggesting that SIRT1 could mediate ferroptosis by activating Nrf2/HO-1 pathway. These results demonstrated that MSCs-Exo ameliorate surgery induced cognitive impairment by inhibiting hippocampus ferroptosis via activating SIRT1/Nrf2/HO-1 pathway.

## 5. Conclusion

In our study, we identified exosomes derived from MSCs and demonstrated that MSCs-Exo can ameliorate cognitive impairment by inhibiting hippocampus ferroptosis via activating SIRT1/Nrf2/HO-1 pathway in dNCR aged mice. These results confirmed that MSCs-Exo may have an efficient medicinal value and provide a novel insight of drug development for dNCR therapy.

## Data Availability

All data generated and analyzed during this study are included in this published article; further inquiries can be directed to the corresponding authors.
